# Conditional survival of pancreatic ductal adenocarcinoma in surgical and nonsurgical patients: a retrospective analysis report from a single institution in China

**DOI:** 10.1186/s12957-015-0608-4

**Published:** 2015-06-06

**Authors:** Rui Liao, Jie Yang, Bao-Yong Zhou, De-Wei Li, Ping Huang, Shi-Qiao Luo, Cheng-You Du

**Affiliations:** Department of Hepatobiliary Surgery, The First Affiliated Hospital of Chongqing Medical University, 400016 Chongqing, China

**Keywords:** Pancreatic cancer, Survival, Prognosis, Surgery

## Abstract

**Background:**

Conditional survival (CS) could offer reliable prognostic information for patients who survived beyond a specified time since diagnosis when the impact of late effects have the greatest influence on prognosis. We aim to investigate CS for pancreatic ductal adenocarcinoma (PDAC) patients with surgery and nonsurgery.

**Methods:**

Chinese PDAC patients between January 2002 and September 2012 were reviewed for analyses. CS rates were calculated for survivors after surgery and nonsurgery at different time points.

**Results:**

Several clinicopathologic features were associated with overall survival (OS) in each subgroup including curative resection, palliative surgery, and nonsurgery. Both univariate and multivariate analyses showed that chemotherapy was a critical predictor for OS regardless of treatment status. CS rates were higher in the curative resected patients than other cases at the same time points. Importantly, stratification of 1-year CS by carcinoembryonic antigen (CEA), (carbohydrate antigen) CA19-9, and tumor stage showed lower CEA, CA19-9, and tumor stage associated with favorable 1-year CS over time (*P* = 0.016, 0.009 and 0.003).

**Conclusions:**

Dynamic CS estimates could be an accurate assessment for the prognosis of PDAC patients, allowing patients and clinicians to project subsequent survival based on time change.

## Background

Pancreas cancer (PC) is a malignant tumor associated with a high mortality rate and is the fourth commonest cause of death from cancer worldwide [1–3]. Pancreatic ductal adenocarcinoma (PDAC) represents about 90 % of histological subtypes of PC and has high relapse rate due to unsatisfying diagnostic technology and management [4]. Over the past decade, therapeutic options for PDAC were still limited. Even after undergoing surgery, chemotherapy or a combination thereof, many patients who have progressed into advanced cancer before clinical manifestation would succumb to tumor recurrence or metastasis. Although the mechanisms through which they cause the disease are still unclear, mounting epidemiological studies have shown that multiple clinical factors such as carcinoembryonic antigen (CEA) and carbohydrate antigen 19-9 (CA19-9) [5–7] were likely to contribute to the increase in the incidence and be related with the prognosis of PDAC [3, 8–10]. But, for the studies on the predictive roles of these clinical factors, most of the investigated patient population were selected from resectable PDAC. Notably, there are still larger portions of patients who underwent palliative surgery without oncologic resection and nonsurgery that owe to lethal malignancy of PDAC. Therefore, stratifying analysis by treatment status on the survival and recurrence of PDAC also needs to be concerned.

However, traditional survival estimates for PDAC could not always be informative enough, or even misleading, for patients who have survived a period of time following treatment. It is increasingly recognized that the chance of survival estimates are dynamic, not a static probability [10–12]. In effect, future survival probability after resection for PDAC is influenced by clinicopathological variables based on the time of diagnosis and surgery and likely to change over time. Many previously published studies [11–13] have also revealed that overall survival (OS) estimates directly applied to survivors of PDAC and it rapidly became inaccurate and irrelevant because of its significant rates of early death and relapse. Conditional survival (CS) estimates have been identified as a practical adjunct to traditional survival analysis, which could offer more accurate and dynamic information of postsurgical patients who have survived longer than expected [14–16]. CS, which calculated the probability of surviving an additional amount of time given that the person has already survived to a predefined period of time after diagnosis and treatment, can update patients’ prognosis [15]. Although CS estimates for patients with PDAC have been reported by several investigations [10–13, 17], we noticed that some important risk factors such as CEA and CA19-9 were not included for CS analyses and most patients were from Europe and United States. Also, to the best of our knowledge, there are currently no published Asian data exploring the CS pattern of PDAC. In this study, we sought to evaluate the CS probabilities of Chinese patients with PDAC stratified by treatment status, which could facilitate to project subsequent survival over time accrued since cancer-directed treatment.

## Methods

### Patient population and data collection

Under the approval of the Ethics Review Committee, between January 2002 and September 2012, 1208 patients with PDAC were selected from the First Affiliated Hospital of Chongqing Medical University. This retrospective cohort study protocol conformed to the ethical guidelines of the 1975 Helsinki Declaration. Written informed consents were obtained from all patients. Among these patients, 380 patients underwent pancreaticoduodenectomy, distal, or total pancreatectomy. For missing curative resection opportunities, 348 and 480 patients were performed by palliative surgical and nonsurgical treatment, respectively. Their minimum survival time is at least 30 days. Patients younger than 18 years and older than 90 years were excluded. Smoking was defined as ≥1 pack/day for more than 10 years [18], and alcoholism was defined as at least 30 g/day of ethanol [19]. The American Joint Committee on Cancer (AJCC) staging system and clinicopathologic data including age, sex, tumor size, tumor grade, tumor location, lymph node status, and so on were assessed. Three senior attending doctors managed the patients who were followed up every 1–6 months postoperatively according to the postoperative time with serum CEA and CA19-9 and abdominal computed tomography (CT) and/or magnetic resonance imaging (MRI).

### Statistic analysis

All statistical analyses were completed with SPSS 19.0 (SPSS, Inc., Chicago, IL, USA) and a two-tailed *P* < 0.05 was considered significant. Results were presented as the mean ± SD. The Student’s *t* test was used for comparison between groups. Clinicopathologic characteristics were analyzed with Fisher’s exact tests, χ^2^ tests, and Spearman ρ coefficients tests. Cumulative event rates and univariate analyses of OS were used by Kaplan-Meier method. Multivariate Cox proportional hazards model was performed to estimate adjusted hazard ratios (HRs) with a 95 % confidence interval (CI). The cut-off values for CEA (range, 0–10 ng/ml) and CA19-9 (range, 0–22U/ml) were determined at two times of upper limit normal value (ULN) in our hospital.

CS was defined as the probability of surviving an additional y years if the patient has already survived x years. Therefore, the formula of CS can be expressed as follows: CS_(y/x)_ = S_(x + y)_/S_x_, where x represents years survived and y stands for additional years [10, 11]. Given the aggressive natural history of PDAC and most of its median, survival after curative surgery is within 1 year [10, 12, 20, 21]; 1-year and 6-month CS for curative resected patients were calculated in the current study. Mathematically, the formula should be expressed as follows: CS_(y/1)_ = S_(1+y)_/S_1_. Similarly, 3-month and 6-month CS were used for analyses of patients with palliative surgery and nonsurgery. Actual survival was the interval from the first surgery to cancer-related death or latest follow-up visit.

## Results

### Baseline characteristics

The baseline characteristics of 1208 Chinese patients with PDAC are described in Table 1. In all, the median age was 63 years (range, 21–88 years) with 736 men and 472 women. The most common presenting symptom was abdominal distention or pain (55 %) followed by jaundice (29.8 %). Few patients have no any symptom (9.9 %) and weight loss (5.3 %). Of the 1208 incident of PDAC cases, some patients had adverse lifestyles and diseases including smoking history (*n* = 448, 37.1 %), alcoholism (*n* = 256, 21.2 %), and diabetes (*n* = 264, 21.9 %). All patients were divided into three subgroups according to treatment status: curative resection, palliative surgery, and nonsurgery. At the time of surgery, 31.5 % (*n* = 380) of patients underwent curative resection including pancreaticoduodenectomy (*n* = 300), distal pancreas (*n* = 64), and total pancreatectomy (*n* = 16). Moreover, 348 cases with palliative surgery did not receive the oncologic resections which have no any influence on their survival. For the patients (*n* = 480) who refused surgery or/and missed the operative opportunities, chemotherapy, radiotherapy, high intensity focused ultrasound, and other nonsurgical treatment had been administered. A substantially higher percentage of patients (73.5 %) were performed with chemotherapy.

**Table 1:** Baseline characteristics of study cohort

Variable	All patients (*n* = 1208)	Surgery		Nonsurgery (*n* = 480)
		curative (*n* = 380)	Palliative (*n* = 348)	
Age (median)	63	61	63	64
Gender (male)	736 (60.9 %)	232 (61.1 %)	248 (71.3 %)	256 (53.3 %)
Smoking (yes)	448 (37.1 %)	148 (38.9 %)	160 (46.0 %)	140 (29.2 %)
Diabetes (yes v no)	256 (21.2 %)	52 (13.7 %)	96 (27.6 %)	116 (24.2 %)
Alcoholism (yes v no)	256 (21.2 %)	80 (21.1 %)	96 (27.6 %)	80 (16.7 %)
Chief complaint				
No symptom	120 (9.9 %)	68 (17.9 %)	8 (2.3 %)	44 (9.2 %)
Abdominal distention/pain	664 (55 %)	172 (45.3 %)	184 (52.9 %)	308 (64.2 %)
Jaundice	360 (29.8 %)	128 (33.7 %)	148 (42.5 %)	84 (17.5 %)
Weight loss	64 (5.3 %)	12 (3.2 %)	8 (2.3 %)	44 (9.2 %)
Tumor characteristics				
Tumor size (≤2)	192 (15.9 %)	80 (21.1 %)	56 (16.1 %)	56 (11.7 %)
Tumor number (single)	1176 (97.4 %)	364 (95.8 %)	340 (97.7 %)	472 (98.3 %)
Tumor encapsulation (yes)	–	284 (74.7 %)	212 (60.9 %)	–
Tumor location (head)	856 (70.9 %)	284 (74.7 %)	340 (97.7 %)	232 (48.3 %)
Tumor differentiation (I–II)	–	276 (72.6 %)	148 (42.5 %)	–
Lymph nodes positive (yes)	–	84 (22.1 %)	96 (27.6 %)	–
AJCC stage (I–II)	512 (42.4 %)	300 (78.9 %)	136 (39.1 %)	76 (15.8 %)
CEA (≤2ULN)	924 (76.5 %)	296 (77.9 %)	292 (83.9 %)	336 (70.0 %)
CA19-9 (≤2ULN)k	316 (26.2 %)	164 (43.2 %)	72 (20.1 %)	80 (16.7 %)
Chemotherapy (yes)	892 (73.8 %)	324 (85.3 %)	308 (88.5 %)	260 (54.2 %)

Tumor differentiation: I, Well differentiated; II, moderately differentiated; III, poorly differentiated; and IV, undifferentiated

*AJCC* American Joint Committee on Cancer staging system, *CEA* carcinoembryonic antigen, *CA19-9* carbohydrate antigen 19-9. *ULN* Upper Limit of Normal. ULN of CEA and CA19-9 are 10 ng/ml and 22U/ml

### Correlation of clinicopathologic features and overall survival

For the whole investigated cohort, median survival of PDAC following treatment was 12.8 months (range, 1–67 months) with an observed 6 months, 1-, 2-, and 3-year survival rates were 64, 42, 27, and 21 %, respectively. Obviously, curative resected cases (21.6 ± 12.2 months) had better prognosis than palliative surgical (10.5 ± 7.5 months) and nonsurgical patients (6 ± 5.5 months). Table 2 presents the results of cox regression analyses by surgical and nonsurgical treatment. Some clinicopathologic features were associated with OS in each subgroup. Importantly, chemotherapy was shown as a critical prognosis related factor after both surgery and nonsurgery. On univariate analyses, CEA and CA19-9 had associations with survival other than age, tumor differentiation, and lymph node status in surgery groups (curative resection and palliative surgery). Tumor stage and tumor size were significant risk factors with the outcome of PDAC after curative resection. Tumor encapsulation and different tumor location correlated with survival of palliative surgical patients. Moreover, CEA, tumor stage, and tumor location also showed predictive value for the outcome of nonsurgical patients with PDAC. Then, competing clinical risk factors were used for further multivariate analyses. Different variables showed significant discrepancy in OS in three subgroups, respectively. For example, poor tumor differentiation and lymph nodes metastasis deem to worsen survival for curative resected patients. While after palliative surgery, older patients, higher CEA, and no tumor encapsulation were independent predictors for OS. In addition, the prognostic abilities of tumor stage and CEA could also be applied to patients with nonsurgical treatment.

**Table 2:** Results of cox regression analyses by surgical and nonsurgical treatment

Factors	Curative resection			Palliative surgery			Nonsurgery		
	Univariate	Multivariate		Univariate	Multivariate		Univariate	Multivariate	
	*P*	HR (95 % CI)	*P*	*P*	HR (95 % CI)	*P*	*P*	HR (95 % CI)	*P*
Age (≤63 v >63)	0.041		0.267	0.002	2.064 (1.222–3.485)	0.007	0.719		NA
Gender (male v female)	0.604		NA	0.411		NA	0.178		NA
Smoking (yes v no)	0.509		NA	0.188		NA	0.902		NA
Diabete (yes v no)	0.976		NA	0.096		NA	0.208		NA
Alcoholism (yes v no)	0.112		NA	0.388		NA	0.723		NA
Tumor size (≤2 v >2)	0.014		0.467	0.283		NA	0.609		NA
Tumor number (single v multiple)	0.143		NA	0.355		NA	0.538		NA
Tumor encapsulation (yes v no)	0.208		NA	<0.001	3.799 (2.147–6.723)	<0.001	NA		NA
Tumor location (head v body/distal/multifocal)	0.116		NA	0.017		0.088	0.021		0.927
Tumor differentiation (I–II v III–IV)	<0.001	4.082 (2.096–7.952)	<0.001	0.017		0.122	NA		NA
Lymph nodes positive (yes v no)	0.001	0.395 (0.196–0.798)	0.010	0.005		0.307	NA		NA
AJCC stage (I–II v III–IV)	0.010		0.764	0.892		NA	0.009	1.405 (1.080–1.828)	0.011
CEA (≤2ULN v >2ULN)	0.013		0.078	0.001	3.568 (1.751–7.269)	<0.001	0.006	1.600 (1.054–2.428)	0.027
CA19-9 (≤2ULN v >2ULN)	0.033		0.339	<0.001		0.066	0.525		NA
Chemotherapy (yes v no)	<0.001	8.050 (2.349–27.591)	0.001	<0.001	13.799 (4.129–45.711)	<0.001	<0.001	5.118 (3.209–8.162)	<0.001

Univariate analysis: Kaplan-Meier method; multivariate analysis: Cox proportional hazards regression model

*CR* curative resection, *IR* incurative resection, *UR* unresection; *Tumor differentiation* I: Well differentiated; II: moderately differentiated; III: poorly differentiated; and IV: undifferentiated, *AJCC* American Joint Committee on Cancer staging system, *CEA* carcinoembryonic antigen; *CA19-9* carbohydrate antigen 19-9, *NA* not adopted

### Conditional survival

Table 3 depicts the results of CS probabilities given that patients have already survived 6 to 36 months in 6-month intervals after treatment. Compared to actual survival, CS probabilities increased over time for the patients in each group whether surgery had been received or not. For example, whereas the actuarial survival at 12 months after curative resection, palliative surgery, and nonsurgery were 77.9, 36.8, and 15.8 %, respectively, the 6-month CS at 6 months, that is postoperative month 6 (to a total of 12), increased to 83.1, 49.3, and 44.1, respectively. Consistent to actual survival, higher CS rates were observed in the patients who underwent curative resection compared to the cases after palliative surgery and nonsurgery at the same time points. Following curative resection, survival at 2 years after surgery was 41.1 % compared with the 12-month conditional survival of 52.8 % after having survived 12 months. Although the 1-year actual survival of the patients after palliative surgery and nonsurgery were very dismal (36.8 and 15.8 %), the 6-month CS at 6 months (to a total of 1 year) still substantially arrived at 49.3 and 44.1 %, respectively.

**Table 3:** Proportion of patients with surgical and nonsurgical pancreatic ductal adenocarcinoma survived for additional time (%)

Total survival^a^	Actual survival (CR/PS/NS)	Time elapsed after surgery and nonsurgery (CR/PS/NS)				
		6 months	12 months	18 months	24 months	30 months
6 months	93.7/74.7/35.8	–				
12 months	77.9/36.8/15.8	83.1/49.3/44.1	–			
18 months	61.1/13.8/5.8	65.2/18.5/16.2	78.4/37.5/36.7	–		
24 months	41.1/4.6/1.7	43.9/6.2/4.7	52.8/12.5/10.8	67.3/33.3/29.3	–	
30 months	22.1/4.6/NA	23.6/6.2/NA	28.4/12.5/NA	36.2/33.3/NA	53.8/NA/NA	–
36 months	20.0/NA/NA	21.3/NA/NA	25.7/NA/NA	32.7/NA/NA	48.7/NA/NA	90.5/NA/NA

*CR* curative resection, *PS* palliative surgery, *NS* nonsurgery, *NA* not adopted for all patients died in the subgroup

^a^If a patient with curative resection has already survived for 6 months, the 6-month conditional survival is 83.1 %

**Fig. 1:**
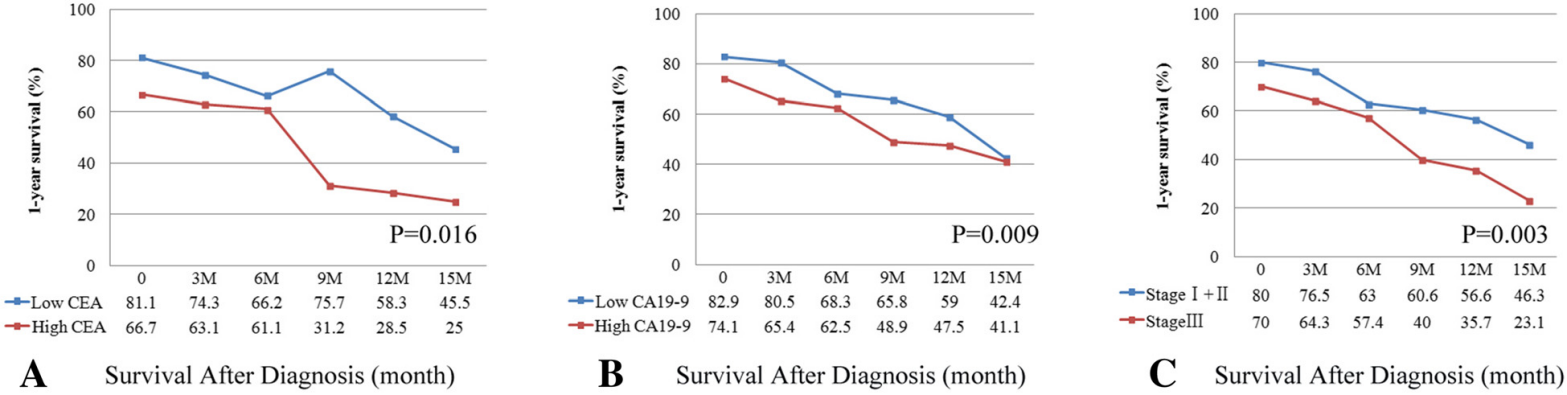
One-year conditional of patients with pancreatic ductal adenocarcinoma (PDAC) after curative resection stratified (CS) by CEA, CA19-9, and tumor stage. **a** One-year CS by CEA (cut-off value, 20 ng/ml); **b** One-year CS by CA19-9 (cut-off value, 44U/ml); **c** One-year CS by stages I through III. The x-axis represents the time after curative resection

To further evaluate the predictive value of some important clinicopathologic features, CS was then stratified by CEA, CA19-9, and tumor stage (Fig. 1) at baseline and 1-year landmark after recurative resection. The patients with lower CEA (*P* = 0.016) and CA19-9 (*P* = 0.009) had higher CS. If the patients have developed into advanced stage (III), those patients who did survive to the 15-month mark, more likely had poorer CS (*P* = 0.003). However, because of the extremely poor prognosis of palliative surgical and nonsurgical patients with PDAC (medians, 8 and 4 months, respectively), CS is not a highly valuable tool to predict the outcome of these patients over time stratifying by lower (stage II and III) and higher (stage IV) stages (both *P* > 0.05).

## Discussion

In the last years, despite tremendous research activities of tumor-specific therapy, PDAC survival is fairly dismal [3]. Hence, analyses of survival outcome and related risk factors have the clinical importance to establish risk nomograms of PDAC which could provide quantitative information for general comparisons and treatment selection. The routine use of CS estimates was strongly recommended by more and more clinicians and researchers because it provided more reliable dynamic prognostic information over time for cancer survivors [10–12]. In this study, we also found PDAC CS probabilities improved over time for all patients based on the additional years already survived by them, no matter what kind of treatment was performed. Furthermore, CS estimates would be more suitable for the patients after curative resection than the population that underwent palliative surgery and nonsurgery due to their very short period of OS, particularly when it was stratified by some clinicopathologic features (e.g., tumor stage, CEA, CA19-9, et al.) for further evaluation of the predictive value of CS.

It is important to know how the chances of long-term survival improve for patients with PDAC after different treatment which can reflect treatment response and outcome from treatment. Predictive accuracy will benefit for our clinical decision-making in a more realistic manner, especially for patients who have survived a period of time beyond surgery or any other nonsurgical treatment. Our study showed that CS estimates could assess the prognosis by dynamic analysis on changing survival expectations of patients with PDAC over time regardless of any treatment. Previously, CS has already been applied in the prognosis estimate of PDAC in several studies [10–13]; however, we found that the majority of patients in the ethnic groups belong to Europe, Australia, and United States, and these investigations focused on the relationship between tumor characteristic and survival of patients with PDAC. For the first time, we analyzed CS of patients with PDAC in Chinese Han population and also explored CS stratified by tumor markers such as CEA and CA19-9. For those patients who underwent curative resection, lower CEA and CA19-9 afforded better CS to the 1-year mark. In fact, the predictive roles of CEA and CA19-9 for OS of PDAC after curative resection have been demonstrated before [5–7]. This may suggest that CEA and CA19-9 derived from cancer cells are involved in the development of PDAC. Therefore, with decreased serum levels of CEA and CA19-9 after tumor remove, CS will continue to improve when time goes on.

PDAC is a very lethal malignancy which is always diagnosed in a symptomatic phase and thus too late to be resected. Several reports about CS of PDAC investigated the patients after curative resection and unresection [10–12], majority of whom belong to stage I and IV. Actually, there still are larger portions of patients who underwent palliative surgery without oncologic resection, whose CS remain to be known. As known, treatment is one of the critical factors to influence CS estimates. Accordingly, the patients in our study were divided into three subgroups by surgical approaches or whether performing operation. For curative resected patients, their CS probabilities and the absolute improvement in CS are much better than those patients with palliative surgery and nonsurgery. On the other hand, compared to nonsurgery subgroup, CS probabilities of patients with palliative surgery are slightly higher (Table 3). These results should be attributed to the temporary improvement of cancer biology after tumor removal, and thus, patients obtain better quality of life. In addition, the purpose of CS calculations is modulation of survival estimates and equation of actual survival by long-term follow-up. At this point, for the patients with palliative surgery and nonsurgery, tumor stage could not accurately reflect their CS by stratify analysis because most of these patients are in advanced stage and have higher recurrence risk and subsequently far shorter survival (<6 months). However, patients with advanced stage would still get better CS than actual survival when they survived for a longer time.

We also found that some adverse lifestyles and disease such as smoking, alcoholism, and diabetes had no relationship with the prognosis of OS, despite that they are important risk factors of PDAC onset and progression [3, 22, 23]. We speculated that after diagnosis and treatment, improvement of diabetes mellitus and quitting smoking and alcohol could benefit the outcome of PDAC. In three subgroups, different clinicopathologic features showed associations with OS. The reason for the discrepancy may be partly due to features of the cancer biology that are different in various tumor stages. Further knowledge about these individual cancer biology features needs studies of mechanism in depth. Notably, our analyses revealed that chemotherapy was shown as a critical prognosis related factor after both surgery and nonsurgery. Many clinicians recommend surgery followed by adjuvant treatment; however, the application of optimal chemotherapy alone or after surgery remains unclear and controversial [24–26]. We believe that comprehensive basic studies and clinic trials on chemotherapy need to be conducted appropriately.

Here, we acknowledge several limitations to the current analyses. The simple size is still far from great. Additionally, for the patients without cancer-directed surgery, we could not obtain the accurate data of tumor differentiation and tumor encapsulation in the absence of a pathologic specimen; also, tumor stage partly depends on the analysis from CT and/or MRI scanning. Indeed, majority of these patients are in advanced stage and have similar survival estimates. Furthermore, our analysis stratifying by treatment status did find the difference of CS. We hope to provide supplementary information for the generalizability of CS application by these investigation results from Chinese Han population of patients diagnosed with PDAC in a single cancer center. From the current study, we could have better understanding for administration of chemotherapy for the prognosis of survival in PDAC. It is not available in Surveillance, Epidemiology, and End Results (SEER) database, although from which we can get large patient population.

## Conclusions

In conclusion, we have shown that CS could provide reliable estimation of future survival for survivors of PDAC after treatment at multiple time points. Moreover, stratifying analyses suggested that CS could be applied in the patients with curative resection more suitably than those treated by palliative surgery and nonsurgery. Also, CEA and CA19-9, related with OS and CS probabilities of the patients with curative resection, should be considered for prognostic nomograms of PDAC. Thus, separate CS estimates by treatment status may be a useful measure to allow clinicians to constantly adjust the treatment for the patients and project subsequent survival based on time change. Furthermore, ongoing study of the use of chemotherapy is required to make. This tool may help us make appropriate decisions for triaging at-risk patients choosing treatment approaches and have significant effects on the quality of life of survivors of PDAC.
